# Association of Operative Day of the Week with the Length of Stay and Total Hospitalization Costs in Patients with Partial Mastectomy: A Nationwide Database Study in Japan

**DOI:** 10.31662/jmaj.2022-0007

**Published:** 2022-06-17

**Authors:** Takaaki Konishi, Michimasa Fujiogi, Nobuaki Michihata, Kojiro Morita, Hiroki Matsui, Kiyohide Fushimi, Masahiko Tanabe, Yasuyuki Seto, Hideo Yasunaga

**Affiliations:** 1 Department of Breast and Endocrine Surgery, Graduate School of Medicine, The University of Tokyo, Tokyo, Japan; 2 Department of Clinical Epidemiology and Health Economics, School of Public Health, The University of Tokyo, Tokyo, Japan; 3 Department of Emergency Medicine, Massachusetts General Hospital, Harvard Medical School, Boston, USA; 4 Department of Health Services Research, Graduate School of Medicine, The University of Tokyo, Tokyo, Japan; 5 Global Nursing Research Center, Graduate School of Medicine, University of Tokyo, Tokyo, Japan; 6 Department of Health Policy and Informatics, Tokyo Medical and Dental University Graduate School Global Nursing Research Center, Graduate School of Medicine, University of Tokyo, Tokyo, Japan

**Keywords:** Breast cancer, Costs, Health service administration, Mastectomy, Length of stay

## Abstract

**Introduction:**

Length of stay (LOS) is a major concern while optimizing medical resources and costs. Hence, factors influencing LOS should be investigated. In Japan, breast cancer surgery generally involves several days of hospitalization for observation, despite few complications. We hypothesized that the day of surgery (weekday; Monday-Friday) affects LOS.

**Methods:**

Using a Japanese nationwide database, we retrospectively identified 146,610 patients who underwent partial mastectomy for stage 0-III breast cancer from July 2010 to March 2017. We conducted multivariable linear and logistic regression analyses adjusting for background characteristics (such as comorbidities and hospital characteristics) with a generalized estimating equation for within-hospital clustering to compare postoperative and total LOS, total hospitalization costs, and postoperative complications between the groups for whom the surgery was performed on different days of the week.

**Results:**

In total, whereas the median postoperative LOS was 4 days (interquartile range, 3-6 days), the median total LOS was 6 days (5-8 days). The median total hospitalization cost was 6,189 US dollars (5,609-6,668 US dollars), and postoperative complications occurred in 3.3% of cases. Despite no significant difference in postoperative complications, Monday-Wednesday surgeries showed significantly shorter postoperative LOS than Friday surgeries (−0.11 days [95% confidence interval, −0.14 to −0.07] on Monday with reference to Friday). Nevertheless, Monday surgeries showed significantly increased total LOS (0.69 days [0.64-0.74]) and hospitalization costs (93 US dollars [71-116]) in comparison with Friday surgeries.

**Conclusions:**

The operative day of the week was associated with increased LOS and cost, with no difference in postoperative complications after partial mastectomy. Surgeries on Monday involved longer preoperative hospital stays and higher total hospitalization costs than those on other weekdays.

## Introduction

Length of stay (LOS) is a major global concern in terms of cost savings ^(1), (2), (3), (4)^, patients’ quality of life ^(5)^, and efficient allocation of hospital resources ^(6), (7)^. Governments and insurers have thus made efforts to decrease LOS to optimize medical costs ^(8), (9), (10), (11), (12), (13)^, and therefore, investigating factors that influence LOS is essential. From a health policy perspective, several socioeconomic factors influence LOS ^(12)^, whereas from a clinical perspective, postoperative LOS is generally associated with the occurrence of complications. A nationwide database study in the US reported that postoperative LOS was associated with age, obesity, comorbidities, surgical procedures, and intraoperative blood transfusion ^(2)^.

Breast cancer is a major malignancy, and mastectomy is the only radical treatment for it ^(14)^. Since mastectomy is seldom associated with severe postoperative complications, it requires shorter LOS than other oncological surgeries ^(15)^; the mean LOS reported in previous Western nationwide studies is 1-2 days ^(15), (16)^. However, since LOS in Japan is generally much longer (approximately three times) than that in other countries ^(17)^, the mean LOS for breast cancer surgery in Japan is approximately 1 week, despite few complications ^(18), (19)^.

Several studies have focused on the operative days of the week; that is, surgery in the latter of weekdays was associated with prolonged postoperative LOS because medical staff shortage on weekends could result in increased complications and lack of rehabilitation ^(20), (21), (22), (23), (24)^. Since clinical problems are irrelevant to breast cancer surgery, we hypothesized that the days of the week when partial mastectomy is performed can be associated with LOS and total hospitalization costs due to nonclinical factors. Using a nationwide database in Japan, this study aimed to examine the association of operative days of the week (Monday-Friday) with postoperative LOS and total hospitalization cost in patients who underwent partial mastectomy for breast cancer.

## Materials and Methods

### Database

This nationwide retrospective cohort study was carried out using the Diagnosis Procedure Combination database, which includes hospital administrative claims data and discharge abstracts for approximately 8,000,000 inpatients in more than 1,200 hospitals throughout Japan each year ^(25)^. The database covers approximately 90% of all tertiary-care emergency hospitals and half of the institutions certified by the Japanese Surgical Society. Participation in the database is mandatory for all 82 university hospitals but voluntary for community hospitals. Because of the anonymity of the patient database, the requirement for informed consent was waived in the present study. This study was approved by the institutional review board of the University of Tokyo [approval number: 3501-(3) (December 25, 2017)].

The Diagnosis Procedure Combination database includes the following data: unique hospital identifier, sex, age, and body mass index at admission; smoking history; main diagnoses and comorbidities at admission and complications after admission recorded as text data in Japanese, using *International Classification of Diseases, Tenth Revision* (*ICD-10*) codes; tumor, node, and metastasis classifications of malignant tumors at admission; interventions and surgical procedures indexed by the original Japanese codes; LOS; and total hospitalization costs. The total hospitalization costs are based on reference prices in the fee schedule that determine item-by-item prices for inpatient services. All discharge abstract data for each patient were recorded at discharge by the attending physicians. Previous validation studies showed good sensitivity and specificity for the diagnosis and procedure records and high validity of cancer diagnosis in the database ^(26), (27)^.

### Study protocol

We retrospectively identified patients who underwent partial mastectomy for breast cancer from April 2012 to March 2017. We used the original Japanese procedure codes for surgery to identify these patients. We excluded patients who (i) underwent axillary dissection, (ii) underwent reconstruction, (iii) had stage IV breast cancer, (iv) underwent surgery on Saturday or Sunday, and (v) had outliers in postoperative LOS (first percentile truncation to avoid analytical problems on an extremely long tail, as in a previous study on LOS ^(28)^). We categorized eligible patients according to the days of the week when the surgery was performed: Monday, Tuesday, Wednesday, Thursday, and Friday.

The primary outcomes were postoperative LOS and total hospitalization costs. We defined the currency exchange rate as 110 Japanese yen per 1 US dollar. The secondary outcomes were the total LOS and postoperative complications. Postoperative complications included surgical site infection (*ICD-10* code: T79.3, T81.4, L02.1), postoperative bleeding (T14.0, T81.0, T81.1, R57.1, R58, N64.5), respiratory complications (J12-J18, J69.0, J80, J95.4-J95.9, J96), urinary tract infection (N10, N12, N30, N39.0), sepsis (A40, A41), heart failure (I21-I24, I50), stroke (I60-I64), acute renal failure (N17), and pulmonary embolism (I26).

We examined the following demographic and clinical characteristics: sex, age, body mass index, smoking history (past/current smoker), comorbidities, preoperative drug use (insulin, oral hypoglycemic drug, and heparin), clinical cancer stage at admission, sentinel biopsy, type of hospital (teaching or nonteaching hospital), and hospital volume. Patients were categorized into six age groups: <40, 40-49, 50-59, 60-69, 70-79, and ≥80 years. Body mass index was categorized into five groups: <18.5 kg/m^2^ (underweight), 18.5-21.9 kg/m^2^ (low normal weight), 22.0-24.9 kg/m^2^ (high normal weight), 25.0-29.9 kg/m^2^ (overweight), and ≥30.0 kg/m^2^ (obese or severely obese). Comorbidities were assessed using the Charlson comorbidity index and categorized as 2, 3, or ≥4 ^(29)^. Hospital volume was defined as the number of breast cancer surgeries performed annually at each hospital and was categorized into tertiles (low, medium, and high) with approximately equal numbers of patients in each group. These background characteristics were adjusted for in the multivariable analyses.

### Statistical analysis

As the main analysis, we performed multivariable regression analyses for outcomes using a generalized estimating equation. We adopted linear regression analysis for continuous outcomes, such as LOS and costs, and logistic regression analysis for postoperative complications. Coefficients and odds ratios for surgeries performed from Monday to Thursday were calculated with reference to surgeries on Friday. We fitted the generalized estimating equation to the analyses to adjust for within-hospital clustering, such as patient characteristics or physician practice patterns within the same hospital.

As a sensitivity analysis, we performed a Poisson regression analysis for LOS without truncation of outliers in postoperative LOS (the exclusion criteria [v]). Poisson regression analysis can allow for analysis considering a long tail in the nonnormal distribution of LOS ^(15), (30)^. Multivariable analysis with the generalized estimating equation was conducted as in the main analysis.

All hypotheses tests had a two-sided significance level of 0.05. We used the Kruskal-Wallis H test to compare medians of continuous variables and the chi-square test to compare proportions of categorical variables. All statistical analyses were conducted using Stata/MP 16.0 (StataCorp, College Station, TX, USA).

## Results

A total of 196,883 patients underwent partial mastectomy for breast cancer from April 2012 to March 2017. We excluded 50,273 patients who met the following exclusion criteria: (i) 46,177 patients underwent axillary dissection, (ii) 1,553 patients underwent reconstruction, (iii) 678 patients had stage IV breast cancer, (iv) 398 patients underwent surgery on Saturday or Sunday, and (v) 1,467 patients had outliers in postoperative LOS ≥ 16 days. Of the eligible 146,610 patients, surgery was performed on Monday for 18,893 patients, Tuesday for 29,313 patients, Wednesday for 32,289 patients, Thursday for 33,088 patients, and Friday for 33,027 patients.

**Figure 1.**
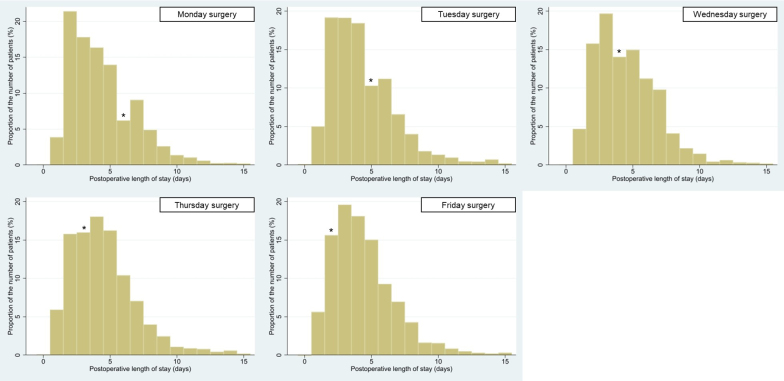
Distribution of postoperative length of stay categorized by surgery day of the week. *denotes Sunday discharge.

Table 1 presents the background characteristics of the patients. Some background factors demonstrated significant but slight differences between the groups for whom surgery was performed on different days of the week. Table 2 shows the crude outcomes without adjustments for such background characteristics. In total, whereas the median postoperative LOS was 4 days (interquartile range, 3-6 days), the median total LOS was 6 days (5-8 days). The median total hospitalization cost was 6,189 US dollars (5,609-6,668 US dollars), and postoperative complications occurred in 3.3% of cases. Figure 1 shows the distribution of postoperative LOS. Patients who underwent surgery on Monday or Tuesday were most often discharged on postoperative day 2, those who underwent surgery on Wednesday, Thursday, or Friday were most often discharged on postoperative day 3 or 4. Additionally, patients who underwent surgery on Monday, Tuesday, or Wednesday were relatively unlikely to be discharged on Sundays.

**Table 1.** Baseline Characteristics of Patients Who Underwent Partial Mastectomy for Breast Cancer Categorized by Surgery Day of the Week.

	Surgery day of the week										
	Monday		Tuesday		Wednesday		Thursday		Friday		
	n = 18,893		n = 29,313		n = 32,289		n = 33,088		n = 33,027		
	n	(%)	n	(%)	n	(%)	n	(%)	n	(%)	P-value
Female sex	18,868	(100)	29,291	(100)	32,260	(100)	33,062	(100)	33,001	(100)	0.24
Age category											<0.001
<40	939	(5.0)	1,384	(4.7)	1,586	(4.9)	1,725	(5.2)	1,730	(5.2)	
40-49	4,351	(23)	6,806	(23)	7,430	(23)	7,891	(24)	8,018	(24)	
50-59	4,060	(21)	6,336	(22)	7,099	(22)	7,244	(22)	7,306	(22)	
60-69	4,993	(26)	7,930	(27)	8,582	(27)	8,694	(26)	8,552	(26)	
70-79	3,251	(17)	4,889	(17)	5,330	(17)	5,458	(16)	5,266	(16)	
≥80	1,299	(6.9)	1,968	(6.7)	2,262	(7.0)	2,076	(6.3)	2,155	(6.5)	
BMI, kg/m^2^											0.019
<18.5	1,646	(8.7)	2,668	(9.1)	2,792	(8.6)	2,858	(8.6)	2,950	(8.9)	
18.5-21.9	6,877	(36)	10,780	(37)	11,754	(36)	12,183	(37)	12,193	(37)	
22.0-24.9	5,410	(29)	8,251	(28)	9,248	(29)	9,276	(28)	9,372	(28)	
25.0-29.9	3,701	(20)	5,800	(20)	6,451	(20)	6,679	(20)	6,376	(19)	
≥30	1,040	(5.5)	1,578	(5.4)	1,734	(5.4)	1,788	(5.4)	1,830	(5.5)	
Missing	219	(1.2)	236	(0.8)	310	(1.0)	304	(0.9)	306	(0.9)	
Past/current smoker	3,488	(18)	5,875	(20)	6,050	(19)	6,912	(21)	6,796	(21)	<0.001
Comorbidities											
CCI											<0.001
2	15,946	(84)	25,260	(86)	27,635	(86)	28,808	(87)	28,402	(86)	
3	2,303	(12)	3,168	(11)	3,477	(11)	3,255	(9.8)	3,658	(11)	
>4	644	(3.4)	885	(3.0)	1,177	(3.6)	1,025	(3.1)	967	(2.9)	
Hypertension	1,631	(8.6)	2,543	(8.7)	2,899	(9.0)	2,723	(8.2)	2,695	(8.2)	0.001
Schizophrenia	71	(0.4)	158	(0.5)	160	(0.5)	176	(0.5)	168	(0.5)	0.11
Preoperative drug use											
Insulin	435	(2.3)	700	(2.4)	746	(2.3)	734	(2.2)	828	(2.5)	0.16
Oral hypoglycemic	435	(2.3)	655	(2.2)	618	(1.9)	675	(2.0)	661	(2.0)	0.008
Heparin	238	(1.3)	278	(0.9)	310	(1.0)	357	(1.1)	339	(1.0)	0.008
Clinical cancer stage											<0.001
0	2,466	(13)	4,144	(14)	4,512	(14)	4,540	(14)	4,905	(15)	
1	11,231	(59)	17,457	(60)	19,685	(61)	19,721	(60)	19,616	(59)	
2	3,835	(20)	5,726	(20)	6,160	(19)	6,572	(20)	6,284	(19)	
3	160	(0.8)	256	(0.9)	262	(0.8)	223	(0.7)	249	(0.8)	
Missing	1,201	(6.4)	1,730	(5.9)	1,670	(5.2)	2,032	(6.1)	1,973	(6.0)	
Sentinel biopsy	15,730	(83)	24,775	(85)	27,700	(86)	28,250	(85)	28,495	(86)	<0.001
Teaching hospital	16,844	(89)	26,587	(91)	32,289	(100)	33,088	(100)	33,027	(100)	<0.001
Hospital volume											<0.001
Low (<77)	5,979	(32)	8,763	(30)	9,106	(28)	10,227	(31)	8,249	(25)	
Medium (78-167)	5,933	(31)	9,336	(32)	13,598	(42)	11,634	(35)	12,181	(37)	
High (≥168)	6,981	(37)	11,214	(38)	9,585	(30)	11,227	(34)	12,597	(38)	

Abbreviations: BMI, body mass index; CCI, Charlson comorbidity index

**Table 2.** Crude Outcomes in Patients Who Underwent Partial Mastectomy for Breast Cancer Categorized by Surgery Day of the Week.

	Surgery day of the week										
	Monday		Tuesday		Wednesday		Thursday		Friday		
	n = 18,893		n = 29,313		n = 32,289		n = 33,088		n = 33,027		P-value	
Postoperative length of stay, days	4	(2-6)	4	(3-6)	4	(3-6)	4	(3-6)	4	(3-6)	<0.001
Total length of stay, days	7	(5-9)	6	(5-8)	6	(5-8)	6	(5-8)	6	(5-8)	<0.001
Total hospitalization costs, US dollar	6,127	(5,639-6,711)	6,058	(5,572-6,629)	6,124	(5,597-6,648)	6,143	(5,642-6,673)	6,137	(5,604-6,692)	<0.001
Postoperative complications	707	(3.7)	815	(2.8)	1,100	(3.4)	1,000	(3.0)	1,234	(3.7)	<0.001

Data are presented as median (interquartile range) or n (%).

**Figure 2.**
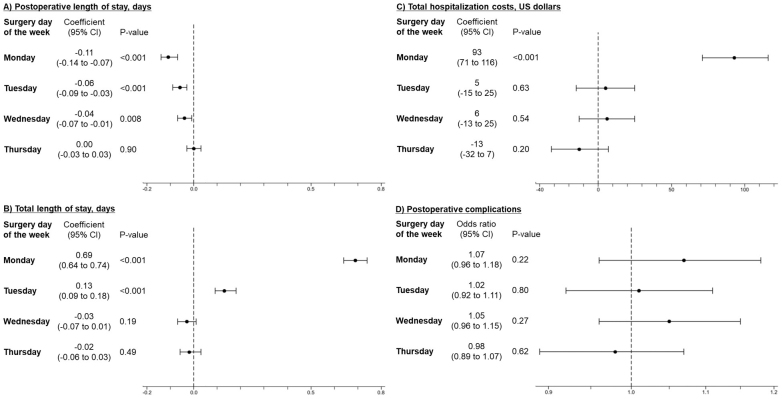
Multivariable linear and logistic regression analysis for outcomes in patients who underwent partial mastectomy for breast cancer. Abbreviation: CI, confidence interval. The coefficients and odds ratios were referenced to the Friday surgery. Multivariable regression analysis with adjustment for background characteristics was conducted using a generalized estimating equation to adjust for within-hospital clustering.

Figure 2 presents the results of the main analysis. Compared with surgeries on Friday, those performed on Monday, Tuesday, and Wednesday were associated with a significantly shorter postoperative LOS (coefficient, −0.11 days; 95% confidence interval [CI], −0.14 to −0.07 days on Monday). However, surgeries performed on Monday were significantly associated with longer total LOS (coefficient, 0.69 days; 95% CI, 0.64-0.74 days) and higher hospitalization costs (coefficient, 93 US dollars; 95% CI, 71-116 US dollars). Postoperative complications did not differ between postoperative days of the week.

Table 3 presents the results of the sensitivity analysis. Poisson regression analyses without exclusion of outliers in postoperative LOS also showed that surgeries on Monday were significantly associated with shorter postoperative LOS but longer total LOS than those on Friday.

**Table 3.** Multivariable Poisson Regression Analysis for the Length of Stay in Patients Who Underwent Partial Mastectomy for Breast Cancer without Exclusion of Outliers in the Postoperative Length of Stay.

	Surgery day of the week											
	Monday			Tuesday			Wednesday			Thursday		
	IRR*	95%CI	P-value	IRR*	95%CI	P-value	IRR*	95%CI	P-value	IRR*	95%CI	P-value
Postoperative length of stay, days	0.98	(0.97 to 0.98)	<0.001	0.99	(0.98 to 0.99)	<0.001	0.99	(0.986 to 0.997)	0.003	1.00	(0.99 to 1.00)	0.82
Total length of stay, days	1.10	(1.09 to 1.10)	<0.001	1.02	(1.02 to 1.03)	<0.001	1.00	(0.99 to 1.00)	0.29	1.00	(0.99 to 1.00)	0.64

Abbreviations: IRR, incidence rate ratio; CI, confidence interval. *IRR is with reference to Friday surgery. Multivariable Poisson regression analysis with adjustment for background characteristics was conducted, using a generalized estimating equation to adjust for within-hospital clustering.

## Discussion

In the present study, we compared LOS and total hospitalization costs in patients who underwent partial mastectomy for breast cancer between groups for whom surgery was performed on different days of the week, using a nationwide database in Japan. The present study revealed that LOS and costs significantly differed between the operative days of the week, despite no difference in postoperative complications. Notably, although postoperative LOS was significantly shorter following surgeries performed on Monday than on Friday, Monday was associated with increased total LOS and hospitalization costs compared with Friday.

A long LOS is a potential concern in Japan ^(11), (12), (13)^. The Organisation for Economic Cooperation and Development reported that the average LOS in acute care hospitals was 5.5 days in the United States, 6.2 days in the United Kingdom, 7.3 days in Korea, and 16.0 days in Japan; that is, Japan demonstrated by far the longest LOS among the reported countries ^(17)^. Such a long LOS in Japan is partly explained by the smaller number of care facilities (e.g., nursing homes) compared with other countries ^(13)^; that is, acute care hospitals often cover postacute care for several days. Regarding postoperative situations, both clinicians and patients would consequently tend to closely monitor surgical wounds and laboratory findings in a hospital setting ^(31)^. A previous study revealed that patients may wish to extend their discharge data even based on superstition (relating to the 6 day lunar calendar that is common in Japan) ^(32)^. Furthermore, doctors could keep patients hospitalized to reduce the number of vacant beds because the payment system based on the Diagnosis Procedure Combination is not on a per-case payment but a per-day payment basis ^(13), (33)^. In Japan, where LOS tends to be extended due to these nonclinical factors, we hypothesized that the day of the week could also affect postoperative LOS, regardless of clinical complications.

Friday can be associated with poor short-term outcomes following surgery, termed as the “Friday effect” ^(24), (34)^. In a study involving 188,212 patients who underwent nonemergent major surgery in the United States, the authors hypothesized that weekend stay in an understaffed regular ward could increase complications after Friday surgery because the first two postoperative days were the most critical for recovery ^(24)^. However, such an association between the day of surgery and complications was not observed in the current study. In breast cancer surgery, which involves little invasiveness and immediate discharge, clinical complications did not increase because of weekend understaffing. Other nonclinical factors may have affected the LOS and hospitalization costs.

Postoperative LOS increased in the latter of weekdays in the present study, despite no significant difference in complications. Some researchers presumed that a lack of rehabilitation might have prolonged postoperative LOS ^(21), (22)^, but such reason would not be applicable in partial mastectomy. Figure 1 shows that patients were unlikely to be discharged on Sunday, although their families could pick them up having to miss work on weekdays. Both doctors and patients may prefer to check the wound on the next weekday (i.e., Monday) rather than discharge on Sunday without a checkup ^(31)^. Because patients who underwent surgery on Monday were discharged during weekdays, their postoperative LOS would not have been affected by the postoperative Sunday effect.

Conversely, total LOS and hospitalization costs increased after Monday surgeries despite short postoperative LOS since patients had prolonged hospital stays before surgery. In Japan, it is common to be admitted to a hospital the day before surgery for orientation and preparation. Because staff shortages on weekends did not allow for sufficient orientation and preparation on weekdays, patients who were scheduled for surgery on a Monday may have been admitted on the previous Friday. The total LOS for Tuesday surgeries was also long despite the short postoperative LOS, presumably due to many national holidays on Mondays in Japan. A long LOS could result in a decreased quality of life and contraction of nosocomial infections ^(5), (32)^. Moreover, the prolonged LOS is not cost-saving ^(1), (2), (3), (4)^; indeed, hospitalization costs increased in Monday surgeries despite a similar occurrence of complications in the current study. We consider that weekend in-advance hospitalization without medical care is common in Japan for any elective procedure, including surgeries scheduled for Mondays. A healthcare system that promotes orientation in the outpatient setting and same-day admission for surgery would be desirable.

The present study only included partial mastectomy without axillary dissection or reconstruction for the homogeneity of the study cohort. The homogeneity allowed for avoiding the effect of invasive procedures on LOS and a simplistic interpretation of the results. Furthermore, we chose partial mastectomy that required hospitalization despite few complications because we aimed to observe the impact of days of the week on LOS and costs regardless of the clinical problems. Although the present study did not show how much the day of surgery affected LOS and costs in other surgeries, we consider the current association to be an inherent and general effect of the Japanese medical system; that is, patients who undergo nonemergency surgery on Monday may be admitted on the previous Friday for orientation and preparation. Therefore, minimizing the impact of Monday surgeries on costs could contribute to a reduction in overall medical expenses.

This study had several limitations. First, since we were able to extract only inpatient data from the database, the number of postoperative outpatient visits was not assessed. Early discharge may cause frequent outpatient visits ^(19)^. Second, we did not include intraoperative blood transfusion, which is known to affect LOS ^(2)^, as an explanatory variable in the analysis. However, in investigating the association of days of the week with LOS and cost, intraoperative blood transfusion was not a confounding variable but an intermediate variable and should not be adjusted for. Third, we were unable to investigate whether patients temporarily stayed out of the hospital during hospitalization. The reason why Tuesday surgeries showed equivalent total costs despite long preoperative hospital stay (presumably due to many national holidays on Mondays) compared with other weekday surgeries might be that patients temporary went out in the daytime or overnight within the three-day holidays (i.e., Saturdays, Sundays, and national holidays on Mondays). Finally, we did not survey patient satisfaction and quality of life in the present study. Avoiding admissions and discharges on understaffed weekends may be justified if it reduces patient anxiety and is cost effective. However, shortened LOS and increased satisfaction have been reported to enhance recovery after surgery programs for several surgeries ^(35), (36)^. We believe that developing a healthcare system that does not extend the LOS due to weekends will improve patients’ quality of life. Moreover, proper allocation of weekend hospital resources could improve the quality of care for other patients ^(3), (6), (7)^ and the optimization of medical costs to maintain the health care system may provide high-quality medical care for patients in the future.

In conclusion, we performed a retrospective study using a large nationwide cohort of patients who underwent partial mastectomy for breast cancer to investigate the association of surgery day of the week with LOS and cost. Despite no significant difference in postoperative complications by the day of surgery, surgeries performed on Monday, Tuesday, and Wednesday were associated with shorter postoperative LOS than those performed on Friday. However, total LOS and hospitalization costs were higher for surgeries performed on Mondays than on other weekdays due to prolonged hospitalization before surgery.

## Article Information

### Conflicts of Interest

None

### Sources of Funding

This work was supported by the Ministry of Health, Labour and Welfare, Japan grant number 21AA2007 and 20AA2005 and the Ministry of Education, Culture, Sports, Science and Technology, Japan grant number 20H03907.

### Author Contributions

Conceptualization: Takaaki Konishi, Michimasa Fujiogi; Methodology: Takaaki Konishi, Michimasa Fujiogi, Nobuaki Michihata; Formal analysis and investigation: Takaaki Konishi, Kojiro Morita, Hiroki Matsui; Writing―original draft preparation: Takaaki Konishi; Writing―review and editing: Takaaki Konishi, Michimasa Fujiogi, Hideo Yasunaga; Funding acquisition: Hideo Yasunaga; Resources: Kiyohide Fushimi, Hideo Yasunaga; Supervision: Masahiko Tanabe, Yasuyuki Seto.

### Approval by Institutional Review Board (IRB)

This study was approved by the institutional review board of the University of Tokyo [approval number: 3501-(3) (December 25, 2017)]. All procedures performed in studies involving human participants were in accordance with the ethical standards of the institutional and/or national research committee and with the 1964 Helsinki declaration and its later amendments or comparable ethical standards.

### Informed Consent

The requirement for informed consent was waived in the present study because of the anonymity of the patient database.

### Disclaimer

Yasuyuki Seto is one of the Editors of JMA Journal and on the journal’s Editorial Staff. He was not involved in the editorial evaluation or decision to accept this article for publication at all.
